# The Role of Salivary Biomarkers in the Early Diagnosis of Alzheimer’s Disease and Parkinson’s Disease

**DOI:** 10.3390/diagnostics11020371

**Published:** 2021-02-22

**Authors:** Patrycja Pawlik, Katarzyna Błochowiak

**Affiliations:** Department of Oral Surgery and Periodontology, Poznan University of Medical Sciences, Bukowska 70 Street, 60812 Poznan, Poland; kasia@naszdentysta.com.pl

**Keywords:** salivary biomarkers, neurodegenerative diseases, Alzheimer’s disease, Parkinson’s disease, α-synuclein, β-amyloid, oxidative stress, TAU

## Abstract

Many neurodegenerative diseases present with progressive neuronal degeneration, which can lead to cognitive and motor impairment. Early screening and diagnosis of neurodegenerative diseases such as Alzheimer’s disease (AD) and Parkinson’s disease (PD) are necessary to begin treatment before the onset of clinical symptoms and slow down the progression of the disease. Biomarkers have shown great potential as a diagnostic tool in the early diagnosis of many diseases, including AD and PD. However, screening for these biomarkers usually includes invasive, complex and expensive methods such as cerebrospinal fluid (CSF) sampling through a lumbar puncture. Researchers are continuously seeking to find a simpler and more reliable diagnostic tool that would be less invasive than CSF sampling. Saliva has been studied as a potential biological fluid that could be used in the diagnosis and early screening of neurodegenerative diseases. This review aims to provide an insight into the current literature concerning salivary biomarkers used in the diagnosis of AD and PD. The most commonly studied salivary biomarkers in AD are β-amyloid_1-42/1-40_ and TAU protein, as well as α-synuclein and protein deglycase (DJ-1) in PD. Studies continue to be conducted on this subject and researchers are attempting to find correlations between specific biomarkers and early clinical symptoms, which could be key in creating new treatments for patients before the onset of symptoms.

## 1. Introduction

Neurodegenerative diseases are characterized by the progressive degeneration of cells of the central and peripheral nervous system, which ultimately lead to cognitive and motor function deficits. Various processes such as oxidative stress, proteotoxic stress and neuroinflammation can induce neuronal degeneration [1,2]. The most common neurodegenerative disorders among the ageing population are Alzheimer’s disease (AD) and Parkinson’s disease (PD), where AD accounts for approximately 80% of all dementia cases [3]. Although PD mainly causes motor deficits, about 30% of all PD cases manifest as full-blown dementia or cognitive impairment [4,5]. The development of dementia in neurodegenerative diseases such as AD and PD begins with mild cognitive impairment (MCI) and increases with age. Deficits of cognitive functions in AD progress from short-term memory impairment, speech deficits and “loss of words” to disturbances in orientation, concentration and attention. In the advanced stages of the disease, symptoms of depression, apathy, sleep disturbance, delusions and hallucinations are observed. Cognitive deficits in PD may precede motor symptoms and include an impairment in planning, abstract thinking, mental flexibility, visuospatial functions, attention as well as memory, and are considered as the main non-motor manifestations of PD [4]. The most characteristic feature of AD and PD is the occurrence of discrete, most often unrecognized neuropathological changes that precede full-blown clinical symptoms. Together with the clinical symptoms they form the basis for the diagnosis and differentiation, as well as the identification of different subtypes of the disease. The well-known neuropathological changes observed in AD are the accumulation of β-amyloid (Aβ) peptides and neurofibrillary tangles (NFTs) in the brain [6]. PD is characterized by the progressive reduction of dopamine levels in the substantia nigra, degeneration of dopaminergic neurons and the formation of intracytoplasmic α-synuclein protein aggregates, known as Lewy bodies, which lead to clinical motor symptoms such as tremors, muscle stiffness, akinesia and bradykinesia, as well as cognitive impairment [7]. These neuropathological changes can commence several years prior to any obvious clinical symptoms, cognitive deficits and memory loss. These clinical observations of AD progression have led to the identification of different AD stages. In the past, the first criteria that addressed the disease described only the later stages, when symptoms of dementia were already evident. According to the updated guidelines, the full spectrum of AD gradually changes over a period of many years. These changes include the preclinical stages of AD, MCI and dementia due to AD. In the preclinical stage, significant clinical symptoms are not yet evident. The MCI stage is characterized by symptoms of memory loss, which are enough to be noticed and measured, but do not compromise the person’s independence. Patients with MCI may or may not progress to AD dementia [8,9,10]. It is estimated that 40 to 60% of MCI patients develop full-blown AD dementia usually many years after the onset of the preclinical stage [11]. Of particular importance is the detection and differentiation between the preclinical and MCI stages so that the diagnosis of AD will not be limited to the diagnosis of dementia due to AD. A similar progression in the disease is seen in PD. Unfortunately, there are no certain diagnostic criteria for the diagnosis of early stages of PD, and most PD patients are correctly diagnosed on the basis of motor symptoms, which are visible when 70% of dopaminergic neurons are lost [12]. The diagnostic frequency of neurodegenerative diseases and accompanying disorders increases with the patients age. Therefore, both AD and PD are mostly diagnosed in elderly people of 65 years and older and are manifested as the last-onset, advanced and fatal neurodegenerative diseases [11]. Delayed diagnosis of AD and PD hinders the implementation of effective therapy and worsens the prognosis. Due to the high prevalence of neurodegenerative diseases among the ageing population, it is important to be able to diagnose and monitor the clinical progression of these diseases at the earliest possible stage. The updated National Institute on Aging and Alzheimer’s Association (NIA-AA) diagnostic criteria for AD distinguish the preclinical and MCI stages of the disease, as well as allow its certain confirmation not only on the basis of an autopsy but also in living patients in the early stages of the disease by means of neuroimaging and biomarker determination [13]. The early diagnosis of AD is based on the identification and analysis of specific biomarkers in the cerebrospinal fluid (CSF) and radiological evaluation using structural or functional magnetic resonance imaging (MRI), as well as Positron Emission Tomography (PET) [11,13,14,15]. These diagnostic methods are not only invasive but time consuming and expensive. PET uses specific tracers to visualize and evaluate Aβ and TAU accumulations in the brain, whereas MRI scans assess function and show brain atrophy, especially in the hippocampus [10]. However, MRI is considered to be reliable only in the later stages of the disease. Another type of imaging modality used in AD diagnosis is ^18^F-2-fluoro-2-deoxy-D-glucose (FDG) PET scans which monitor glucose metabolism mechanism and identify areas of decreased brain activity [13]. According to the assumptions of the introduced diagnostic guidelines, biomarkers obtained from CSF are to help in the identification of the early stages of the disease and in the assessment of the disease progression. However, their use as a diagnostic method is clinically limited due to insufficient standardization of the analytical results, limited availability and a lack of evidence correlating biomarker concentration with AD pathology. In AD, all biomarkers are classified into an A/T/N system, in which A represents Aβ concentration, T refers to TAU levels, and N includes neurodegeneration and neuronal injury biomarkers [13]. To sum up, according to the NIA-AA, diagnosis of AD on the basis of biomarkers is based on the determination of a reduced level of Aβ_1-42_ and Aβ_1-42_/ Aβ_1-40_ ratio in CSF or the detection of Aβ aggregates on PET scans, as well as increased TAU levels in CSF and its aggregates detection on PET scans. Currently, only the detection of TAU and Aβ in CSF or alternatively their aggregates on PET scans are considered reliable in the diagnosis of AD. Attempts are being made to determine these biomarkers in other body fluids as an alternative to CSF or to search for other biomarkers specific to AD, as well as to differentiate its different stages. Similar attempts to identify and introduce biomarkers into diagnostics were carried out in PD. Due to its presence in the subarachnoid space and ventricular system of the brain and spinal cord, as well as reflecting pathological changes in the brain, CSF is a natural source of diagnostic biomarkers in neurodegenerative diseases. However, the CSF sampling is an invasive procedure which involves pain, risk of complications, and is unsuitable for frequent repetition in routine practice. Hence, the continuous search for the use of biomarkers derived from other peripheral body fluids. Blood has also been suggested as a diagnostic tool, considering that it is safer than a lumbar puncture and less invasive. However, studies have shown that AD-specific biomarkers in blood are difficult to isolate due to their low concentration, which would require a highly sensitive technical modality [14]. Moreover, AD is comorbid with vascular risk factors, thus, the presence of these variables may affect the results obtained [13]. Researchers have been focused on finding an alternative, less invasive and more affordable diagnostic tool that would allow to identify specific biomarkers in neurodegenerative diseases at an early stage. Moreover, these biomarkers could be helpful in the monitoring of disease progression and therapy effectiveness, as well as in the identification of different subgroups in AD and PD. Easy accessible biomarkers could be used as a screening tool in the most predilected patients [11,15,16].

Saliva is an alternative biological fluid that has been widely used as a diagnostic material in areas such as toxicology, infectious diseases, endocrinology and cardiology [17,18]. Some salivary proteins have also been used in the identification of neurologic and psychiatric disorders [2,19]. Saliva plays an important function in the protection and maintenance of healthy oral mucosa and teeth through its buffering capacity and its antibacterial and antiviral properties. It can be treated as an equivalent of serum. Saliva is a suitable biomaterial that can be used as a diagnostic method because it is relatively easy to obtain, the procedure is non-invasive, its processing is simple, it possesses lower protein content than blood and urine, and is less expensive [20,21,22]. A summary of the main advantages and disadvantages of saliva as a biological fluid in the diagnosis of neurodegenerative diseases when compared to other biological fluids such as CSF and blood is presented in Table 1. 

Additionally, salivary flow and composition are regulated by the autonomic nervous system, which would suggest that a direct relationship between saliva and the nervous system exists and that specific biomarkers linked to neurodegenerative diseases can be detected in saliva. When it comes to obtaining saliva from patients, the preferred method is passive drooling of unstimulated saliva where whole saliva is collected into a tube. This method is considered the golden standard as it is easy and non-invasive due to the fact that both salivary flow and composition are not affected, thus allowing analytes in saliva to be quantified without any changes [23]. Even though this method is considered the golden standard, it may become challenging with some dementia patients that are unable to cooperate either physically, which is the case in PD or psychologically, as is the case in advanced AD. In cases where the passive drooling method cannot be applied, sampling of saliva by absorption is the preferred method [19,24]. Once saliva sampling is completed, salivary biomarkers can be determined by various methods such as enzyme-linked immunosorbent assay (ELISA), western blot, immunofluorescence, flow cytometric assays, multiplex array assays, infrared (IR) spectroscopy, chromatography mass spectrometry, and Ellman colorimetric method [19,25]. The divergent results in research studies on the use of salivary biomarkers are mainly due to the use of different research methods and their different levels of sensitivity. However, when comparing the same biomarkers using different methods, they show a similar trend, not always demonstrating statistical significance, which indicates the need to determine the sensitivity of each method for salivary markers.

The studies that were included in the presented review were identified using PubMed and Google Scholar. The databases were searched using specific keywords such as: “neurodegenerative disease”, “Alzheimer’s disease”, “Parkinson’s disease”, “salivary biomarkers”, “dementia”, “saliva”, “biomarkers”, “alpha-synuclein”, “beta-amyloid”, “TAU”, “oxidative stress” in various combinations. We included literature written in English and published between the years of 1999 and 2020. 

Despite the significant progress in the search for biomarkers that could play an important role in the early diagnosis of neurodegenerative diseases, this review aims to describe and introduce novel biomarkers that can be isolated not only from CSF but also from saliva. Based on the well-known and specific biomarkers used for the diagnosis of AD and PD in CSF, we aim to present clinical studies that have isolated and compared these AD and PD-specific biomarkers in saliva. We present the current state of knowledge on the possibility of determining markers in saliva with a recognized diagnostic role as an alternative to CSF. We pay attention to saliva-specific biomarkers, which so far have not been analyzed in CSF or in blood, as well as indicate the possibility of determining new neurodegenerative markers in saliva, the levels of which are significantly changed in CSF or in the blood as a new direction in saliva research. The aim of this review is to provide an extensive insight into recent clinical studies where salivary biomarkers were isolated and used in the screening, differentiation between various stages and subtypes, and early diagnosis of AD and PD, as well as to present the limitations and future approaches of salivary biomarkers as a diagnostic tool in the diagnosis of AD and PD.

## 2. Biomarkers in the Diagnosis of Alzheimer’s Disease

AD is an etiologically and clinically heterogeneous neurodegenerative disease, which is associated with the progressive death of cholinergic neurons within the hippocampal and cortical regions, the consequence of which is atrophy, abnormal neurotransmission and loss of synapses. The pathophysiology behind the development and progression of AD involves various biochemical and molecular mechanisms. At the molecular level, the underlying mechanisms of AD involve the extracellular pathogenic deposition of Aβ peptides and the intracellular formation of hyperphosphorylated TAU protein aggregates in the form of NFTs, which lead to the degeneration of neurons and their synapses, the activation of glial cells, oxidative stress, and chronic neuroinflammation [26,27,28]. 

The source of Aβ plaques, the pathological accumulation of which underlies AD, is the incorrect cleavage of the amyloid precursor protein (APP). In physiological conditions, APP is cleaved by α-secretase into soluble APP alpha (s-APPα) and an 83 AA fragment (C-83), which is then further cleaved by γ-secretase into p3 peptide and the APP intracellular domain (AICD) [2,29]. Studies have shown that APP plays an important function in brain homeostasis and is involved in neural growth and maturation during brain development [30,31]. In AD, instead of being cleaved by α-secretase, APP is cleaved by β-secretase, also known as BACE-1, and γ-secretase. This enzymatic cleavage cascade results in the formation of amyloid beta 40 (Aβ_1-40_) and 42 (Aβ_1-42_) peptides, which accumulate and form plaques in the extracellular space, causing neuronal toxicity and inducing a reactive inflammatory process that ultimately leads to neuronal damage [2,28,32,33]. This amyloidogenic pathway is a well-known source of diagnostic biomarkers for AD. Detection of Aβ deposition through PET scans and Aβ levels in CSF, as well as in other body fluids are used as diagnostic methods of AD. Among the various Aβ isoforms, the levels of Aβ_1-40_ and Aβ_1-42_ are the most reliable for AD diagnosis. Specifically, Aβ_1-42_ aggregates into plaques within the brain and its concentration in CSF is reduced, which serves as an indicator for AD. Although Aβ_1-40_ is the most abundant isoform, there are no significant changes in its levels in AD patients. In this case, its levels are analyzed by the Aβ_1-42_/Aβ_1-40_ ratio, which is more reliable than only assessing single Aβ_1-42_ or Aβ_1-40_ concentrations due to individual fluctuation compensations [28]. Moreover, other truncated forms of the Aβ_1-42_ amyloidogenic peptides which include Aβ_37,_ Aβ_38,_ Aβ_39_ could provide additional diagnostic value. The accuracy of the Aβ_1-42_/Aβ_1-38_ ratio is comparable to that of Aβ_1-42_/Aβ_1-40_ ratio in predicting AD. Most of the Aβ isoforms are widely distributed in the CSF, as well as in other body fluids and peripheral tissues and may be used as AD biomarkers. However, the diagnostic levels of these isoforms can be different due to disease heterogeneity, co-morbidities, assay specificity and sensitivity, sampling differences, and body fluid processing and storage. In order to increase diagnostic accuracy of Aβ, its levels are analyzed in combination with TAU isoforms. TAU is another protein involved in the pathophysiology of AD. TAU is a microtubule-associated protein that is involved in the stabilization of microtubules in the cell [2]. This stabilization is important when it comes to proper neuronal structure and axonal transport in neurons [34]. In AD, mutations in the TAU protein sequence alter the phosphorylation site, which leads to excessive phosphorylation of TAU, which in turn leads to an increased accumulation of NFTs and consequently neuronal death [2,35,36]. Increased levels of total TAU (t-TAU) and phosphorylated TAU (p-TAU) in the CSF are characteristic for neurodegeneration. The decrease in Aβ_1-42_, and concomitant increase in Aβ_1-42_/Aβ_1-38_ and Aβ_1-42_ / Aβ_1-40_ ratios, as well as t-TAU and p-TAU levels is commonly referred to as an AD profile [28,37]. 

The diagnosis of AD is accomplished by using various diagnostic tools, which when all put together give an accurate and reliable diagnosis. One of the diagnostic methods used to assess AD-specific biomarkers is through CSF sampling. Due to its direct relationship with the nervous system, CSF sampling is considered the most sensitive and specific (specificity around 90-95%) for the early detection of AD-specific biomarkers such as Aβ_42_, p-TAU and t-TAU [38,39]. In order to diagnose AD, the value of Aβ_1-42_ in the CSF should be decreased by 50%, and there should be a significant increase of 200% of p-TAU and a 300% increase of t-TAU [40]. Although the measurements of Aβ_1-42_, t-TAU and p-TAU in the CSF, as well as the visualization of fibrillary Aβ protein loads in the brain using a radioactive ligand have proven useful in the diagnosis of AD and have been included in the diagnostic guidelines, independent new biomarkers are sought mainly for monitoring the disease progression and assessing the response to treatment [41]. This is due to a weak correlation between the concentration of Aβ in the CSF or amyloid PER uptake and the disease severity. The TAU protein is better correlated with the clinical picture, however, its diagnostic value decreases with the advancement of neurodegeneration. Moreover, neither the Aβ nor TAU protein alone reflect the severity and progression of cognitive impairment. The probability of developing dementia is diverse in terms of the presence of Aβ and TAU pathology [42]. Therefore, alternative non-Aβ and non-TAU biomarkers are being evaluated. Potential new biomarkers in the diagnosis of AD and PD are Aβ and TAU independent proteins associated with various pathological processes occurring in neurodegenerative diseases such as neuroinflammation, axon degeneration, synaptic loss, vascular disorders, iron toxicity and lipid metabolism disorders. Their disadvantages are the lack of specificity for AD and PD and their occurrence in advanced stages of neurodegenerative diseases. Increased concentrations of Neurofilament Light Polypeptide (NLP) and neurogranin in the CSF and blood correlates with the degree of cognitive impairment and may be useful in the prognosis of the development of cognitive disorders in AD. Another marker protein for neuronal damage is visinin-like protein 1 (VILIP-1), the increased concentration of which in the preclinical phase and MCI predicts future cognitive decline. Other candidate biomarkers related to neurodegeneration are chromogranin-A and secretogranin-1, which are characterized by elevated concentrations in MCI and decreased concentrations in dementia. Markers of neuroinflammation and inflammatory cell activation are postulated as potential biomarkers in the diagnosis of AD. These include progranulin, soluble Triggering Receptor Expressed on Myeloid cells 2 (sTREM2), chitinase-3-like protein 1 (YKL-40) and interferon-γ-induced protein 10 (IP-10). They are associated with microglia activation and their increased levels in AD correlate with an overall development of dementia and brain cortical atrophy in the future. Combinations of subsets of new biomarkers enhance their utility in terms of broadly characterizing AD-associated pathological changes for comprehensive monitoring of the treatment response and for precise selection of susceptible patients [8,43]. Although these markers were determined only in the CSF and in the blood, it seems that their identification in saliva could be a valuable supplement in diagnostics. At the moment, the most reliable diagnostic method for diagnosing AD is through biomarker analysis of the CSF and neuroimaging, however, researchers are moving forward and attempting to find new biomarkers in other biological fluids such as saliva. All AD biomarkers can be divided into AD-specific biomarkers group, the determination of which in CSF is included in the current diagnostic criteria, and into non-AD- specific biomarkers group, the level of which differs significantly in the AD group compared to controls. They are related to neurodegenerative processes and can be regarded as candidate biomarkers. They require further research into their usefulness in the diagnosis of cognitive disorders, the differentiation of AD from other neurodegenerative diseases, and the detection of early AD stages. In addition, biomarkers specific for certain body fluids, including saliva, are distinguished, the level of which is changed in AD compared to the control group. This indicates new directions of research on biomarkers in AD. Table 2. presents AD-specific biomarkers, as well as other potential biomarkers and candidate biomarkers that can be isolated in CSF, blood and saliva. 

### Salivary Biomarkers in the Diagnosis of Alzheimer’s Disease 

AD-specific salivary biomarkers that have been studied and quantified include Aβ_1-40_, Aβ_1-42_, p-TAU, t-TAU and lactoferrin. The diagnostic use of the salivary Aβ_1-40_, Aβ_1-42_ levels in AD were based on the presence of Aβ protein deposits in peripheral regions, including skin, nasal mucosa, lacrimal and lingual glands, in addition to the classic accumulation in the brain. Moreover, salivary gland biopsies have been described as a tool for research on familial amyloidotic polyneuropathy and AD because APP and Aβ are expressed in salivary epithelial cells. In a study conducted by Lee et al. the authors reported that Aβ_1-42_ is continuously produced in the body by not only the brain but all other organs. They collected saliva and tissue samples from different organs including the spleen, kidneys, hippocampus, brain, small intestine and pancreas from 10 patients with severe AD and 27 healthy participants. The Aβ_1-42_ level in healthy participants was approximately 20 pg/ml, while in patients with AD or at risk of developing AD, the level was double (40 pg/ml). The authors did not report any significant differences in Aβ_1-42_ concentration when comparing different stages of the disease [43]. Sabbagh et al. isolated salivary Aβ_1-42_ using the same methodology as Lee et al. from 15 patients with AD and eight healthy participants. Their results were similar to those of Lee et al. in that there was a significant increase in the level of Aβ_1-42_ in AD patients compared to healthy participants. Moreover, AD patients had a 2.45-fold increase in Aβ_1-42_ compared to the control [44]. A study conducted by Bermejo-Pareja et al. investigated the levels of Aβ_1-42_ and Aβ_1-40_ in saliva of 70 AD patients, 51 PD patients and 56 healthy participants. The authors not only focused on determining the levels of Aβ_1-42_ and Aβ_1-40_, but also on assessing the correlation between the concentration of Aβ_1-42_ and the severity of AD. The results obtained showed that the level of Aβ_1-42_ in saliva was higher in AD patients when compared to PD and healthy participants, however, this difference was not significant. A significant increase in salivary Aβ_1-42_ level was observed in patients with mild and moderate AD when compared to patients with severe AD and healthy participants. Moreover, the increased Aβ_1-42_ salivary levels in AD were independent of AD risk factors, including age and Apo E genotype. In conclusion, the results of this study showed that the level of Aβ_1-42_ is specific to AD patients and not other neurodegenerative diseases such as PD [45]. In addition to its importance in the diagnosis of AD and its differentiation from other neurodegenerative diseases, Aβ can be used in the diagnosis of early stages of the disease, diagnosis of cognitive disorders, and assessment of disease severity and progression. Kim et al. conducted a study where the levels of salivary Aβ were correlated with the severity of AD. The authors evaluated and compared Aβ_1-42_ and Aβ_1-40_ levels in 28 AD patients that were categorized as having severe or mild cognitive impairment (MCI) to 17 healthy participants without any neuropathological symptoms or cognitive impairment. Unlike in the other studies where ELISA kits were used, the authors of this study used antibody-based magnet nanoparticles immunoassay. The results showed a significant increase in the Aβ_1-42_ levels in patients with severe AD in comparison to healthy participants [46]. This was not the case in previous studies conducted by Bermejo-Pareja et al. where levels of Aβ_1-42_ in patients with severe AD were significantly lower when compared to patients with mild and moderate AD [45]. Kim et al. also compared the ELISA method to the facile microarraying method, which used antibody-based magnet nanoparticles immunoassay to identify salivary Aβ_1-42_ levels. They observed that the results obtained from the antibody-based magnet nanoparticles immunoassay had a higher accuracy rate for the identification of low concentrations of Aβ_1-42_ in saliva than the ELISA method [46]. A similar study assessing the AD progression was conducted by McGeer et al. who revealed lower salivary Aβ_1-42_ level in low-level control group of AD development risk compared to the high-level control group of AD development risk. Division of study groups was based on the immunohistochemical post-mortem assessment of Aβ_1-42_ accumulation in the brain of AD patients. In the low-level control group salivary Aβ_1-42_ level was remarkably constant between the age of 16-92. Moreover, salivary Aβ_1-42_ level in AD patients was greater than the high-level control group. These data demonstrated that measuring salivary Aβ_1-42_ levels can diagnose AD and indicate as well as that it may predict the risk of future onset [47].

Most studies have focused on the quantification and detection of Aβ_1-42_ in saliva, however, some researchers took it upon themselves to attempt to quantify other biomarkers such as p-TAU, t-TAU and t-TAU/p-TAU ratio. Both Aβ_1-42_ and TAU protein levels in the CSF and in other body fluids have been used either alone or in a combination in AD diagnosis. Similar to Aβ and APP, TAU proteins are expressed in salivary epithelial cells [48]. The probable source of the TAU proteins in saliva are the nerves innervating the salivary glands and the acinar epithelial cells. Salivary TAU levels directly or indirectly reflect the pathological changes in AD salivary glands and the brain. A study conducted by Shi et al. attempted to quantify t-TAU, p-TAU and Aβ_1-42_ levels in saliva taken from both 21 AD patients and 38 healthy volunteers by using Luminex assay. Moreover, they detected five unique TAU peptides in saliva using mass spectrometry. The authors observed that mass spectrometry did not allow for the quantification of Aβ_1-42_, however, there was a significant increase of the t-TAU/p-TAU ratio in AD patients. Their results suggested that salivary TAU may be shifted toward a phosphorylated form that is essential to disease development and progression in AD patients. Furthermore, contrary to increased CSF levels of t-TAU and p-TAU in AD, salivary t-TAU level is unchanged or headed in an opposite direction. Salivary p-TAU level is much higher than t-TAU within the same object [49]. One of the reasons for the higher salivary p-TAU levels is the preferential secretion of p-TAU by the salivary glands and the effect of stimulation of salivary secretion on the increased concentration of p-TAU. Pekeles et al. attempted to quantify the t-TAU/p-TAU ratio by using western blot analysis. The authors used saliva samples from 46 AD patients, 55 MCI patients and 47 healthy participants in order to quantify t-TAU/p-TAU ratio on different phosphorylation sites. The results showed a significant increase in the t-TAU/p-TAU ratio in AD patients compared to both MCI and healthy participants. However, these results did not correlate with results obtained from CSF samples. When CSF was used, there was no significant differences in p-TAU/t-TAU ratio when comparing AD, MCI and healthy patients [50]. 

Another salivary biomarker that has been studied in the diagnosis of AD is lactoferrin. Lactoferrin is abundantly present in saliva and plays a role in the modulation of immune reactions and inflammation. It has been shown that lactoferrin has Aβ-binding properties and therefore, could play an integral role in the pathophysiology of AD [2]. It is an antimicrobial peptide synthesized mostly by glandular epithelial cells [17]. A recent study conducted by Carro et al. focused on investigating lactoferrin as an AD salivary diagnostic biomarker. They divided their subjects into four groups: patients with AD, patients with amnestic mild cognitive impairment (aMCI), patients with PD, and healthy participants with no cognitive impairment. Using sodium dodecyl sulphate-polyacrylamide gel electrophoresis (SDS-PAGE) fractionation and mass spectrometry analysis, the authors were able to analyze lactoferrin levels and found that both in the aMCI and AD group of patients, there was reduced levels of lactoferrin when compared with the control group of healthy participants. These results were then confirmed by ELISA analysis. In order to confirm that the low levels of lactoferrin were specific to AD, the authors decided to measure lactoferrin levels in saliva of PD patients. The results showed that salivary lactoferrin concentrations were higher in patients with PD compared to the control group of healthy participants. What’s interesting is that 78% of healthy participants from the control group that presented with lactoferrin concentration of below 7.43 μg/mL converted to an aMCI or AD diagnosis within 5 years. This suggests that lactoferrin could be used as a precise biomarker that could help in identifying patients that suffer from AD or aMCI at earlier stages of the disease. Moreover, salivary lactoferrin levels positively correlated with mini-mental state examination (MMSE) scores and Aβ_1-42_ level, and negatively correlated with t-TAU level [17]. To assess the diagnostic utility of salivary lactoferrin in AD patients, González-Sánchez et al. examined the relationship between salivary lactoferrin and cerebral Aβ load using Amyloid-Positron-Emission Tomography (PET) neuroimaging in two different cross-sectional cohorts including 52 healthy asymptomatic subjects considered as controls, 21 MCI due to AD, 25 with AD dementia and 18 with frontotemporal dementia (FTD). One hundred and forty-two participants, including 74 healthy subjects and 68 MCI, composed cohort 2. Additionally, 39 subjects from the MCI group were diagnosed with prodromal AD and the others as MCI not due to AD, which was based on the amyloid-PET results. Of all the control participants, four subjects from cohort 1 and four subjects from cohort 2 had positive amyloid-PET results. In two cohorts, salivary lactoferrin levels were significantly lower in MCI-PET positive and AD groups compared to FTD patients and cognitively healthy subjects from cohort 1, healthy controls and MCI-PET negative patients from cohort 2. There were no differences in salivary lactoferrin levels between MCI-PET positive and AD patients and between controls and MCI-PET negative groups. These results revealed that decreased salivary lactoferrin levels are AD-specific biomarkers and are helpful in differentiating and diagnosing the early clinical stages of AD, as well as in predicting the development and progression of cognitive disorders in AD [51]. 

Acetylcholinesterase (AChE) has also been suggested as a potential marker in the diagnosis of AD. AChE is an enzyme that plays a role in the breakdown of acetylcholine (ACh), which is released into the synaptic cleft during a neuronal impulse. Its diagnostic use in AD is based on the decreased concentration of acetylcholine (ACh) caused by the degeneration of cholinergic neurons and the significant defect in cholinergic conductivity observed even in the initial stage of AD [52]. A decline in cholinergic function is closely correlated with loss of memory, cognitive and learning impairment in AD patients. It is postulated that AChE levels are an indicator of the state of cholinergic neurons. AChE is widely distributed not only in the nervous system and CSF, but also in peripheral tissues, muscles and other body fluids such as blood and saliva. Moreover, cholinergic neurons are responsible for salivary secretion, making salivary AChE a valuable diagnostic marker. According to Bakhtiari et al. salivary AChE activity evaluated by using the Ellman colorimetric method in 15 AD patients was lower than that of the control group which included 15 participants. However, there were no statistically significant differences in the enzyme activity between these groups and there were no correlations between AChE activity and age, gender and the duration of the disease [53]. The different results were received by Ahmadi-Motamayel et al. who reported a significant increase of salivary AChE and pseudocholinesterase (PChE) activity in the AD group compared to the healthy subjects. This increase did not correlate with the disease duration, however, there was a negative correlation between enzyme activity and age [54]. A decreasing trend in the activity of AChE in the AD group compared to the controls was found by Boston et al. The study group was composed of 15 AD patients, 13 healthy controls and 13 patients with vascular dementia. However, detected changes were not statistically significant [55]. In order to reduce the negative effect of cholinergic conduction defect, acetylcholinesterase inhibitors (AChE-I) are used in the treatment of AD, which help to increase and prolong the activity period of the released acetylcholine. Sayer et al. assessed salivary AChE levels in the study group which included 36 AD patients (22 AD responders to AChE-I and 14 AD non-responders to AChE-I) and 11 healthy subjects. This study revealed a decreased salivary AChE activity in AD patients compared to the controls. In addition, AChE was significantly decreased in the AChE-I non-responder group compared to the responder group [56]. Moreover, changes in synapse function and neurotransmission indicate the potential use of neurotransmitters other than acetylcholine in diagnosing AD. In a study by Peña-Bautista et al., salivary myo-inositol and creatine levels were significantly lower in AD compared to the control group, while acetylcholine levels were higher in the AD group compared to controls. There were no differences in salivary taurine, aspartic acid, glutamic acid, glutamine, γ-aminobutyric (GABA), N-acetyl-L-aspartic acid and acetonitrile levels between AD and healthy controls. Salivary levels of myo-inositol, creatine, glutamine and acetylcholine correlated with some cognitive tests scores. Furthermore, a multivariate analysis including sensitivity, specificity and area under the curve (AUC) revealed a few promising indices for creatine, acetylcholine, glutamine and myo-inositol. These neurotransmitters could be used as promising non-invasive biomarkers for diagnosing AD and cognitive impairment [57].

Another possible cause of neurodegeneration is oxidative stress, defined as the imbalance between the production of reactive oxygen species (ROS) and the efficiency of enzymatic and non-enzymatic defense systems, which include catalase, superoxide dismutase, glutathione peroxidase and antioxidants. Oxidative stress leads to mitochondrial dysfunction, neuroinflammation and the accumulation of neurotoxic proteins. There is a close relationship between excessive production of ROS and the accumulation of Aβ in peripheral tissues and organs such as salivary glands [58]. The local redox imbalance in salivary glands is responsible for the impairment of the structure and function of salivary glands. It is postulated that oxidative stress is a key factor in causing xerostomia in patients with different types of dementia. Therefore, salivary oxidative stress biomarkers could be valuable and helpful in the diagnosis of AD and different types of dementia, as well as cognitive impairment. In a study by Choromanska et al., 80 patients with moderate dementia and 80 healthy age- and sex-matched individuals were studied. The salivary uric acid levels, catalase and peroxidase activity were significantly lower in dementia patients compared to the controls. Moreover, in both non-stimulated and stimulated saliva, mean total oxidant status (TOS) and oxidative stress index (OSI) values in the dementia group were higher than those in the control group. The mean total antioxidant capacity (TOC) values in the dementia group were lower than those in the control group. This study revealed increased salivary levels of the DNA products, protein and lipid oxidative damage with simultaneous reduction of saliva secretion in dementia patients. The detected markers of oxidative damage, which included 8-isoprostanes, 8-hydroxy-2’-deoxyguanosine, advanced glycation end products, advanced oxidation products and advanced glycation end products (AGE), were indicative of a very high diagnostic value in the diagnosis of dementia [58]. Similar signs of depletion of antioxidant defense systems in saliva in dementia patients were detected by Klimiuk et al. where the study group was composed of 26 patients with mild to moderate dementia, 24 patients with severe dementia and 50 healthy participants. Superoxide dismutase, catalase and glutathione peroxidase activity in saliva in patients with dementia was decreased compared to the control group. Moreover, reduced glutathione salivary levels (GSH) were decreased in patients with severe dementia compared to those with mild to moderate dementia. These results indicated that salivary GSH may clearly distinguish patients with different severity of dementia [59]. 

Other potential AD biomarkers are saliva metabolites. Liang et al reported increased levels of spinganine-1-phosphate, ornithine and phenyllactic acid, and decreased levels of inosine, 3-dehydrocarnithine and hypoxanthine in AD patients compared to healthy participants by using the fast ultraperformance liquid chromatography mass spectrometry (FUPLC-MS) [60,61]. In a study conducted by Huan et al, there were statistically significant differences in salivary levels of methylguanosine, histidylphenylalanine, choline-cytidine, phenylalanyproline between AD patients and healthy participants when using liquid chromatography mass spectrometry. Moreover, there were differences in salivary phenylalanylproline and alanylphenylalanine levels between AD and the MCI group [62]. Finally, increased levels of trehalose in AD patients compared to the control group were found in a study by Lau et al, however, these results were not statistically significant [63].

Many salivary biomarkers have been explored and studied when it comes to diagnosing neurodegenerative diseases, however further studies are needed in order to find the proper diagnostic tools for the early diagnosis and the progression of neurodegenerative diseases. A detailed summary of all the described potential salivary biomarkers associated with AD are presented in Table 3.

## 3. Parkinson’s Disease

PD’s pathophysiological mechanism is characterized by a progressive loss of dopaminergic neurons, which leads to an overall reduction in dopamine levels in the brain, as well as increased levels of cytoplasmic α–synuclein inclusions known as Lewy bodies [64]. Unlike in AD, data related to the identification and possible use of biomarkers in the diagnosis of PD are limited. There are no biomarkers validated for the diagnosis of idiopathic PD, which is the form that occurs in 90% of cases. Changes in the concentration of any substance are not included in the diagnostic criteria of PD. Moreover, there are no reliable biomarkers that could help in the correlation of neurodegeneration with clinical features and to distinguish PD from atypical parkinsonism. The diagnosis of PD is performed using single-photon emission computed tomography (SPECT) with the radiotracer imaging of dopaminergic transporter (DAT) and brain PET. However, a definitive confirmation usually requires pathological examination during autopsy, where progressive degeneration of dopaminergic neurons in the substantia nigra and Lewy bodies formation in surviving neurons are observed. The Unified Parkinson’s Disease Rating Scale (UPDRS) is used for the assessment of the mental and physical conditions in PD. The search for biomarkers for the early diagnosis of PD is currently the focus of many researchers. The most promising marker is α–synuclein. Moreover, in the familial form of PD, accounting for 10% of all PD cases, patient’s autosomal dominant and recessive mutations in the α–synuclein gene (SNCA) are detected. The use of α–synuclein relies on its rich expression in the central nervous system and its misfolding leading to the formation of an oligomeric form, which is responsible for Lewy bodies and Lewy neurites development [65,66]. It plays a role in modulating the stability of the neuronal membrane and membrane trafficking through vesicular transport. Furthermore, it accounts for up to 1% of total protein in cytosolic brain fraction. α–synuclein exists in four different isoforms, which have different aggregating potential and various risks of abnormal aggregation. Some factors such as oxidative stress, proteolysis, fatty acid concentration, phospholipids and metal ions can modulate the structure of α–synuclein, leading to alternative formations of the protein, including oligomeric forms, which can develop into cytoplasmic inclusions. Additionally, post- translational modifications such as phosphorylation can also result in altered protein size. Phosphorylated α–synuclein is involved in the development of Lewy bodies and it is reported that phosphorylation at the Ser-129 site is characteristic of PD and related to synucleinopathies. Therefore, α–synuclein and Lewy bodies are markers of other neurodegenerative disease termed α–synucleinopathies, which include PD with or without dementia, Lewy body variant of AD, multiple system atrophy, and dementia with Lewy bodies. For differentiation of these neurodegenerative diseases additional diagnostic tools should be used. Moreover, its levels are used for the diagnosis of non-motor symptoms related to mainly cognitive PD dysfunction. α–synuclein has been thus far identified in solid tissues as well as in CSF, plasma and saliva [67]. In general, total α–synuclein in the CSF of PD patients is lower independently on the used laboratory methods, showing a high predictive value. The oligomeric and phosphorylated α–synuclein levels were significantly increased in the PD group [68]. It seems that its levels in CSF are PD-specific and sensitive marker. On the other hand, α–synuclein levels in the blood, especially in red cells are elevated. The high fragility of red cells could result in the possible contamination of CSF. Therefore, the quantification of α–synuclein in saliva could be a valuable diagnostic method for PD diagnosing [34]. Saliva is easily accessible and free of blood contamination. Goldman et al. examined the relationship among CSF, plasma and saliva α–synuclein levels in PD patients and healthy controls. Contrary to previous findings, the reported no differences in plasma and saliva α–synuclein levels between PD and the control group. Moreover, there were no significant correlations for α–synuclein between CSF and plasma, CSF and saliva or plasma and saliva. Additionally, there was a correlation between α–synuclein levels in CSF and selected motor and non-motor PD symptoms and UPDRS scores, only. No similar correlation was detected for salivary and plasma α–synuclein levels [68]. Another biomarker potentially involved in PD pathology is protein deglycase-1 (DJ-1). It is associated with the early onset of familial autosomal recessive PD. It is postulated to be a pleiotropic neuroprotective protein. Additionally, it plays a role as an antioxidant and against mitochondrial dysfunction. DJ-1 can be active in the inhibition of the formation of α–synuclein fibrils [2]. In addition to α–synuclein and DJ-1, attempts to test the usefulness of other biomarkers, mainly related to neurodegeneration and oxidative stress in PD diagnosis are being made.

### Salivary Biomarkers in the Diagnosis of Parkinson’s Disease 

The diagnostic use of salivary α-synuclein is based on finding its presence in nerve fibers innervating salivary glands and comparing the concentration of the oligomeric and monomeric forms of α-synuclein. Moreover, submandibular gland biopsies presented with positive staining for α-synuclein in PD patients, which provided strong evidence for the use of saliva as a source in diagnosing PD biomarkers [69]. Apart from salivary glands, α-synuclein can be identified in salivary exosomes. The oligomeric α-synuclein (α-syn _olig_) and α-syn _olig/_ α-syn _total_ ratio in salivary exosomes were higher in PD than in controls, however, there were no correlations between α-syn _olig_ and α-syn _olig/_ α-syn _total_ ratio and the disease duration and UPDRS score [70]. Results from previous studies related to α-synuclein levels in saliva are conflicting, showing either an increase in salivary α-synuclein in PD patients compared to the control groups or no alternation in salivary α-synuclein levels [34,71]. The first study conducted by Al-Nimer et al. reported a lower salivary level of α-syn _total_ in PD patients than in healthy controls. However, they did not take into account the contribution of different isoforms to the total α-synuclein level. They quantified the total α-synuclein levels in saliva samples of 20 PD patients and 20 healthy subjects [71]. Vivacque et al. detected the oligomeric and total α-synuclein in the saliva of 60 PD patients and 40 healthy patients using ELISA assay. They reported a significant decrease in salivary total α-synuclein (α-syn _total)_ levels in PD patients compared to healthy controls. Conversely, salivary oligomeric α-synuclein (α-syn _olig_) levels were higher in PD patients than in healthy participants. Accordingly, the α-syn _olig/_ α-syn _total_ ratio was significantly higher in PD patients than in healthy controls This shift in both proportions is due to the axonal and intracellular aggregation of the oligomeric form in PD. Moreover, a positive correlation was reported between α-syn _total_ levels and disease duration, as well as UPDRS total score. A negative correlation was found between the Montreal Cognitive Assessment score and α-syn _total_ levels [65]. These results suggest that the evaluation of salivary α-syn _total_ concentration may be a helpful tool in the diagnosis of PD, particularly in the early stages of the disease. Similar findings were confirmed by the same authors in a larger study group that included 112 PD patients, 90 healthy controls and 20 patients with progressive supranuclear palsy (PSP). They detected decreased salivary α-syn _total_ levels in PD patients compared to the healthy controls and significantly increased salivary α-syn _olig_ levels in PD patients compared to the control group, as well as an increase in the α-syn _total_ / α-syn _olig_ ratio. Moreover, α-syn _total_ concentration in PSP patients was found to be significantly higher compared to PD patients and the control subjects [66]. Contrary to the previous study, there were no correlations between α-syn _olig,_ α-syn _total_ or α-syn _total_ / α-syn _olig_ ratio and the disease duration and the UPDRS score in PD patients as well as in PSP patients. These results revealed that salivary α-synuclein can differentiate PD patients from PSP patients and that salivary α-synuclein is a PD-specific biomarker. The potential relationship between salivary α-synuclein levels and α-synuclein gene (SNCA) was studied by Kang et al, where 201 PD patients and 67 healthy controls were investigated. There was no significant difference in saliva α-synuclein levels between PD patients and controls, as well as between males and females. Moreover, its levels did not correlate with the UPDRS score. Salivary α-synuclein levels decreased with age in PD patients but not in healthy controls. Salivary α-synuclein levels were closely associated with genotypic distribution of rs11931074 and rs894278 in the PD group. Moreover, α-syn _olig/_ α-syn _total_ ratio increased with disease progression. These results suggest that salivary α-syn _olig_ levels might be a potential biomarker for disease progression monitoring of PD patients. G allele of rs11931074 was correlated with lower salivary α-syn _total_,, while G allele of rs894278 was correlated with higher levels of salivary α-syn _total_ [72]. A cohort study of 25 patients with PD and 15 HC subjects was conducted by Shaheen et al., where the total and oligomeric forms of salivary α-synuclein were quantified and correlated with disease severity. The results obtained showed an increase of the total α-synuclein/oligomeric α-synuclein ratio in PD patients compared to HC subjects, and a decrease of total α-synuclein in salivary samples. However, there was no significant correlation between the total α-synuclein concentration and disease severity [73]. Some research has focused on isolating and quantifying the DJ-1 protein. A study conducted by Devic et al. used Western blot analysis to quantify total α-synuclein and DJ-1 from the saliva of 24 PD patients and 25 HC subjects, as well as evaluated the correlation between these proteins and the severity of PD. The results obtained showed lower levels of total α-synuclein in PD patients compared to HC subjects. However, there was a slight increase in salivary DJ-1 levels in PD patients compared to HC subjects. The total α-synuclein and DJ-1 levels did not show any correlation to the UPDRS scores [74]. Another study focused on the quantification of total proteins, DJ-1, amylase, albumin and mucins from the saliva of 16 PD patients and 22 HC subjects by using ELISA assay. The authors of this study showed an increase in the levels of total proteins, amylase, albumin and DJ-1 protein in the saliva of PD patients compared to HC subjects. There was no significant difference between the levels of mucins in saliva of both PD patients and HC subjects [75]. Moreover, the adjusted DJ-1 levels correlated with disease severity measured by using the Movement Disorders Society-UPDRS (MDS-UPDRS). These results also suggested that the saliva of PD patients is different in composition. Contrary to the previous studies, Kang et al reported no correlations between salivary DJ-1 levels and UPDRS scores. Moreover, the same authors revealed a close relationship between salivary concentrations of DJ-1 and putamen nucleus uptake of the labeled dopamine transporters in SPECT, which provided evidence for the use of DJ-1 as a biomarker of nigrostriatal dopaminergic function in PD and an adjuvant or alternative diagnostic tool. Its level correlated with PD severity because salivary DJ-1 levels were higher in patients with stage 4 in the Hoehn and Yahr (H&Y) scale than those with stages 1-3 in the H&Y, as well as those in healthy controls. These results indicated that salivary DJ-1 levels could be a valuable biomarker for monitoring disease progression. Furthermore, DJ-1 levels may help to differentiate various PD subtypes including tremor dominant type, akinetic-rigid dominant type and mixed type. Its level was significantly decreased in the mixed type of PD patients compared to other PD types [76].

Other than the direct relationship between DJ-1 and familial type of PD there is an indirect involvement of DJ-1 in PD onset and progression by the oxidative stress pathomechanism. Oxidative stress can change the DJ-1 cell localization and favor its mitochondrial or nucleus translocation. Under low or moderate oxidative stress, DJ-1 plays a neuroprotective function as it has the ability to reduce hydrogen peroxide species and oxidative stress, as well as to regulate the expression of antioxidant proteins [2]. One of the postulated biomarkers of oxidative stress involved in PD is heme-oxygenase-1 (HO-1), which is an indicator of the body’s adaptive response to increased levels of ROS in patients with PD [77]. Song et al. compared salivary HO-1 levels in 58 PD patients with the different disease severity and 59 healthy controls. They reported significantly higher HO-1 concentrations in saliva of PD patients relative to the controls. Its levels correlated with the H&Y scores and were higher in the early stage of PD than in PD patients with stage 2 and stage 3. Its levels were independent to age, sex, comorbid illnesses and medication exposure [78].

The use of salivary AChE as a biomarker in PD results from the observation that xerostomia and decreased salivation is a concomitant symptom of the disease. Dopaminergic neuron loss in PD is accompanied by loss of cholinergic neurons and this deficit is more severe in patients affected by PD-related dementia [2]. In a study by Fedorova et al. PD patients presented significantly decreased salivary flow rate, significantly increased salivary AChE activity and total protein (TP) concentration compared to controls. AChE levels should be combined with the total protein levels. The AChE/TP ratio was significantly higher in PD patients than in controls. However, AChE levels and AChE/TP ratio did not correlate with the UPDRS scores. Furthermore, there were correlations between AChE salivary activity and different stages of PD assessed by the H&Y scores [79]. A summary of all the described potential salivary biomarkers associated with PD are presented in Table 4.

Future studies must be conducted to further analyze if levels of salivary total α-synuclein, oligomeric α-synuclein, DJ-1 protein, and other potential markers can be used as salivary biomarkers not only to detect PD but to also determine the severity and differentiate between the various stages of PD.

## 4. Limitations of Salivary Biomarkers

The diagnosis of AD and PD has been a challenge for many practitioners for many years. As opposed to the current diagnostic methods, which are invasive and expensive, many researchers have started studying saliva as a potential diagnostic tool for the diagnosis of neurodegenerative diseases such as AD and PD. The presented results of research on the use of various salivary markers in the diagnosis of AD and PD revealed significant differences, which prove the need for their further verification on larger and more homogeneous groups at similar stages of the disease due to the fact that some markers significantly change their concentration along with the disease progression. Determining the concentration in homogeneous groups will allow the elimination of different endotypes in AD and PD, and the identification of biomarkers exclusively associated with specific disease subgroups or type of disorders. Saliva is highly accessible and can be collected non-invasively, which makes it a great diagnostic tool. However, the kits used for salivary biomarkers have not yet been standardized, which makes it difficult to make a specific diagnosis. To standardize the study of salivary markers and introduce their concentration into routine diagnostics, it is necessary to determine the sensitivity and specificity of the markers. The method of saliva collection and its type are of significant importance in the study of salivary markers. The stimulation of salivary secretion significantly changes its composition, which is reflected in the concentration of saliva-specific biomarkers and proteins. Moreover, salivary content may be influenced by the circadian rhythm and time of sample collection [80]. The stimulation of salivary flow also changes the percentage of individual salivary glands, leading to a change in the content of salivary proteins. Another challenge with using saliva as a diagnostic tool is that even though it is highly accessible, clinical symptoms of AD and PD can prevent the proper collection of saliva due to poor compliance from the patient. A common symptom in PD is hypersialorrhea as well as xerostomia, both of which may hinder the collection of saliva. Saliva in patients with hypersialorrhea and xerostomia has a different consistency and concentration. In order to eliminate the effect of hyposalivation, parameters evaluated in saliva should be standardized for total protein content or salivary flow rate. Moreover, many drugs that affect the central nervous system such as anxiolytics, neuroleptics and tricyclic antidepressants can interact with the cholinergic muscarinic receptors and reduce salivary flow. Other drugs used in systemic diseases including antihypertensive, analgesics, antihistamines and chemotherapeutics may also change the qualitative composition of saliva and reduce the salivary flow. Although saliva is the equivalent of serum, it reflects local changes in the mouth induced by bacteria, hygiene habits and exogenous factors. The oral cavity is extremely exposed to many harmful environmental factors such as tobacco smoke, ethanol, drugs and dental materials. This applies primarily to oxidative stress markers, which reflect the delicate balance between local salivary antioxidants and ROS. Periodontitis and oral mucosa disorders can trigger oxidative stress. Another limitation results from low protein concentration and high intra-individual and inter-individual variability. Except for saliva sampling, the other factors such as its storage, handling, processing and analysis technique are of great importance. Lack of standardization for these procedures limits the use of saliva as a diagnostic method for AD and PD.

## 5. Future Perspectives in the Use of Salivary Biomarkers 

Despite significant progress in the identification and analysis of markers used in the diagnosis of AD and PD, the results of research on salivary markers are still very limited and require confirmation in larger study groups. This mainly concerns biomarkers with a well-known diagnostic value and widespread and determined in other body fluids. The second direction of research is the search for biomarkers that can differentiate between the various stages of the disease and can be useful in its monitoring and assessment of its progression. Research on biomarkers allowing for early diagnosis of the preclinical and MCI phase of AD, as well as predicting disease progression from the preclinical phase and MCI to the development of dementia are of particular importance. In addition to markers with a recognized diagnostic value in AD, research into the use of salivary-specific proteins such as lactoferrin and new markers such as iron- and Aβ-binding glycoproteins should be conducted. The existence of 366 proteins and peptides with a potential diagnostic role in AD is postulated [48]. In addition, sialometric tests can help in the early detection of dementia, as decreased salivation may be one of the first symptoms of dementia. Reduced salivation can induce changes in the composition of salivary proteins while emphasizing changes in concentrations specific for dementia and neurodegenerative diseases. Sialometric tests are of particular importance in PD. Future studies are also looking at the microbiome in the oral cavity and salivary exosomes [48,81]. A new study by Rani et al. explored a novel method that directly correlates salivary exosomes concentration with the progression of cognitive impairment in AD. The authors used nanoparticle tracking analysis (NTA) in order to quantify and analyze salivary exosomes. The results showed that the concentration of salivary exosomes was significantly higher in patients with cognitive impairment and AD compared to healthy participants. Fluorescent antibody based quantification of surface marker exosome CD63 was used to validate the results obtained from the NTA method. In this method, salivary exosomes are used as a progression marker that could allow researchers to not only detect the specific concentrations but to correlate them with the progressive loss in cognitive functioning [82]. There is growing evidence of a close relationship between the oral microbiome and the progression of AD and it has been observed that as cognitive function declines, oral health also deteriorates [83,84]. A study showed that AD patients had increased circulating levels of tumor necrosis factor-alpha (TNF- α) and antibodies for oral bacteria such as *P. gingivalis* in serum compared to healthy participants, which shows that there is a direct relationship between the oral microbiome and AD [85]. In a study conducted by Poole et al., *P. gingivalis*-derived lipopolysaccharides were found to be present in brain samples taken from patients with AD [86]. It can be observed that a link between oral pathogens found in the oral microbiota and AD exists and further studies would have to be carried out in order to standardize and evaluate the specific relationship between the oral microbiome and AD. Another new area of research on salivary biomarkers in AD and PD are proteins reflecting pathological processes in neurodegenerative diseases such as IP-10, VILIP-1, YKL-40, TREM-10, NFL, neurogranin and synaptotagmin. They require verification of their specificity for AD and PD and confirmation of potential determination of their levels beyond CSF and blood, as well as confirmation of their usefulness in saliva. Research on the diagnostic use of salivary metabolites and autophagic- and lysosomal-related biomarkers such as cathepsin D, glucocerebrosidase and heat-shock cognate protein (HSC70) is also indicated. They are exclusively associated with PD or AD [87].

## 6. Conclusions

Many researchers have studied the potential use of saliva as a diagnostic tool in the diagnosis and early screening of neurodegenerative diseases, in particular AD and PD. Contrary to CSF, saliva is a body fluid that can be non-invasively collected, is readily accessible and provides the possibility of obtaining many samples. Studies conducted have shown that salivary biomarkers can be quantified and used to diagnose AD and PD. With further investigation and standardization of the collection and quantification methods, as well as larger sample groups, salivary biomarkers could become the golden standard in the diagnosis and early screening of AD and PD.

## Figures and Tables

**Table 1:** Summary of advantages and disadvantages of saliva as a biological fluid in the determination of biomarkers compared to CSF and blood.

	CSF	Blood	Saliva
**Advantages**	- Standardized methodology	- Easily accessible and non-invasive collection technique - Inexpensive - Reproducible results - Aβ can bypass through the blood-brain barrier	- Equivalent to serum - Easily accessible and non-invasive collection technique - Inexpensive - Chair-side procedure, does not require hospitalization - Reproducible results at different stages - Lower stress during collection for patients with neurodegenerative diseases
**Disadvantages**	- Invasive collection technique (lumbar puncture) - Expensive - Requires hospitalization and specialized clinicians	- Non-specific methodology - Low concentration of biomarkers	- Non-specific, non-standardized methodology

**Table 2:** Detailed presentation of both AD biomarkers and candidate biomarkers and their levels comparison between CSF, blood and saliva.

	Biomarker	CSF	Blood/Plasma	Saliva
**AD specific biomarkers (Aβ and TAU)**	Aβ isoforms:			
	Aβ_1-42_	↓	Inconsistency	↑
	Aβ_1-40_	Inconsistency	Inconsistency	Unchanged
	Aβ_1-38_	Inconsistency	Inconsistency	No data
	Aβ_1-42_/Aβ_1-40_ ratio	↓	↓	No data
	Aβ_1-42_/Aβ_1-38_ ratio	↓	No data	No data
	TAU			
	t-TAU	↑	↑	↓
	p-TAU	↑	↑	↑
**AD non-specific biomarkers** **Non-Aβ and non-TAU biomarkers**	Inflammation/neuroinflammation biomarkers:			
	TREM2	↑	No change	No data
	YKL-40	↑	↑	No data
	IP-10	Inconsistent	Inconsistent	No data
	ICAM1	↑	No data	No data
	Synaptic dysfunction biomarkers:			
	Neurogranin *	↑	No change	No data
	SNAP-25	↑	No data	No data
	Synaptotagmin	↑	↓ (limited data)	No data
	Secretogranin-2	↓	No data	No data
	Neuronal pentraxin 1	↓	No data	No data
	Neurofascin	↓	No data	No data
	Myelin basic protein	↑	No data	No data
	BACE1	↑	↑	No data
	α-Synuclein	↑	No change	No data
	TDP-43	No data	↑	No data
	Lactoferrin	No data	No data	↓
	Acetylcholinesterase	No data	No data	↓
	Neuronal injury biomarkers:			
	NFL	↑	↑	No data
	VILIP-1	↑	↑	No data
	Iron toxicity biomarkers:			
	Ferritin	↑	No change	No data
	hFABP	↑	No change	No data

↓: decreased level in AD compared to controls; ↑: increased level in AD compared to controls; TREM2, triggering receptor expressed on myeloid cells 2; YKL-40, Chitinase-3-like protein 1; IP-10, interferon-γ-induced protein 10; ICAM1, intercellular adhesion molecule 1; *, potentially high degree of AD specificity; SNAP-25, synaptosome-associated protein 25; BACE1, β-site amyloid precursor protein cleaving enzyme 1; TDP-43, transactive response DNA-binding protein 43; NFL, neurofilament light polypeptide; VILIP-1, visinin-like protein 1; hFABP, heart-type fatty acid-binding protein.

**Table 3:** Potential salivary biomarkers associated with Alzheimer’s disease (AD) described in clinical studies.

Potential. Biomarker	Cohort (*n*)	Methods	Results	References
**A-β_42_**	AD: 10 with severe AD	ELISA assay	↑Aβ_42_ in AD, no significant difference in stages of disease	Lee et al. [43]
	AD: 15 with mild to moderate AD HS: 8	ELISA assay	↑Aβ_42_ in AD than in HS (AD patients have a 2.45-fold increase)	Sabbagh et al. [44]
	AD: 70 (29 mild, 24 moderate and 17 severe) PD: 51 HS: 56	ELISA assay	↑ Aβ_42_ in AD than in PD and HS but not statistically significant ↑ Aβ_42_ in mild and moderate AD ↑ Aβ_42_ in mild AD vs HS *p* = 0.043	Bermejo-Pareja et al. [45]
	AD: 28 HS: 17	Antibody-based magnet nanoparticles immunoassay	↑ Aβ_42_ in severe AD vs. HS, ↑ Aβ_42_ in severe AD vs. MCI	Kim et al. [46]
	AD: 21 HS: 38	Luminex assay	Undetectable	Shi et al. [49]
	AD: 23 Low controls: 25 High controls (risk for AD) 6	ELISA assay	↑ Aβ_42_ in AD compared to high controls and low controls, AD > high controls > low controls	McGeer et al. [47]
**A-β_40_**	70 AD (29 mild, 24 moderate and 17 severe) PD: 51 HS: 56	ELISA assay	Unchanged expression between AD, PD, and HS group	Bermejo-Pareja et al. [45]
**t-TAU**	AD: 21 HS: 38	Luminex assay	Trend for ↓ t-TAU in AD compared to HS	Shi et al. [49]
**p-TAU**	AD: 21 HS: 38	Luminex assay	Trend for ↑ p-TAU in AD compared to HS	Shi et al. [49]
**p-TAU/t-TAU ratio**	AD: 21 HS: 38	Luminex assay	↑significantly in AD	Shi et al. [49]
	AD: 46 MCI: 55 HS: 47	Western Blot analysis	↑significantly in t-TAU/p-TAU ratio in AD vs. MCI and HS	Pekeles et al. [50]
**Lactoferrin**	AD: 80 MCI (amnestic MCI): 44 PD: 59 HS: 80	ELISA assay	↓ lactoferrin in AD and MCI compared to HS ↑ lactoferrin in PD compared to HS	Caro et al [17]
	1 cohort: 116 MCI-PET^+^: 21 AD dementia: 25 FTD -PET: 18 HS: 52 (4 PET^+^, 48 PET^-^) 2 cohort: 142 HS (cognitively normal): 74 (4 PET^+^ and 70 PET^-^) MCI: 68 (39 MCI-PET^+^ due to AD, 29 MCI-PET^-^ not due to AD)	ELISA assay	↓ lactoferrin in MCI-PET^+^ and AD compared to HS and FTD ↓ lactoferrin in MCI-PET^+^ compared to HS and MCI-PET^-^ No differences between HS and MCI-PET^-^ ↑ lactoferrin in the PET^-^ group compared to the MCI-PET^+^ group	González-Sánchez et al. [51]
**Acetylcholinesterase (AChE)**	AD: 15 HS: 15	Ellman colorimetric method	↓ AChE in AD vs. HC, no significant difference in enzymatic activity, no correlation between AChE, age, disease progression	Bakhtiari et al. [53]
	AD: 30 HS: 30	Ellman colorimetric method	↑ AChE and and PChE in AD	Ahmadi-Motamayel et al. [54]
	AD: 15 HS: 13 VD: 13	Ellman colorimetric method	↓ AChE in AD	Boston et al. [55]
	AD: 36 (22 responders to AChE-1; 14 non-responders) HS: 11	Ellman colorimetric method	↓ AChE in non-responders vs. responders	Sayer et al. [56]
	MCI due to AD: 17 Mild to moderate dementia AD: 14 HS: 12	Chromatography mass spectrometry	↓ significantly myo-inositol and creatine levels in AD vs. HS, AChE ↑ in AD, no differences in taurine, aspartic acid, glutamic acid, glutamine, GABA, N-Acetyl-L-aspartic acid, acetonitrile	Peña-Bautista et al. [57]
**Oxidative stress markers**	Dementia: 80 (moderate stage) HC: 80	Redox assay, antioxidant assay (spectrophotometry method)	↓ salivary uric acid, catalase, peroxidase in dementia, ↑ TOS and OSI in dementia, ↑ salivary levels of DNA products, protein and lipid oxidative damage	Choromanska et al. [58]
	Dementia: 50 (AD-dementia: 15; VD: 19; mixed dementia: 16) HS: 50	Redox assay, antioxidant assay (spectrophotometry method)	↓ in superoxide dismutase, catalase, glutathione peroxidase activity in patients with dementia, ↓ glutathione salivary levels (GSH) in patients with severe dementia	Klimiuk et al. [59]
**Saliva metabolomics**	AD: 256 HS: 218	Fast ultra-HPLC coupled with TOF-MS	↑ sphinganine-1-phosphate, ornithine, phenyl lactic acid in AD patients compared to HS ↓ inosine, 3-dehydrocarnitine, hypoxanthine in AD patients compared to HS	Liang et al. [60]
	Discovery Phase group: MCI: 25, HS: 35, AD: 22 Validation Phase group: MCI: 10, HS: 10, AD: 7	Differential chemical isotope labelling liquid chromatography mass spectrometry	Statistically significant difference in methylguanosine, histidylphenylalanine, cholinecytidine, phenylalanyproline between AD and HS, difference between phenylalanylproline and alanylphenylalanine between AD and MCI	Huan et al. [62]
	AD: 20 PD: 20 HS: 20	ELISA assay	↑ trehalose in AD vs. HS (not significant)	Lau et al. [63]

↑: increasing; ↓: decreasing; VD: AD subgroup with vascular dementia; TOS: total oxidant status; OSI: oxidative stress index.

**Table 4:** Potential salivary biomarkers associated with Parkinson’s disease (PD) described in studies.

Potential Biomarker	Cohort (n)	Methods	Results	References
Total α-synuclein	PD: 20 HS: 20	ELISA assay	↓ total α-synuclein in PD	Al-Nimer et al. [71]
	PD: 60 HS: 40	ELISA assay	↓ total α-synuclein in PD	Vivacque et al. [65]
	PD: 112 HS: 90 PSP: 20	ELISA assay	↓ total α-synuclein in PD, ↑ total α-synuclein in PSP vs. PD	Vivacque et al. [66]
	PD: 201 HS: 67	Luminex assay Western blot Magnetic bead- based luminex assay	No significant difference between PD and HC, no correlation with UPDRS score, ↓ with age in PD but not HC, associated with specific g-alleles	Kang et al. [72]
	PD: 25 HS: 15	ELISA assay	↓ total α-synuclein in PD	Shaheen et al. [73]
Oligomeric α-synuclein	PD: 60 HS: 40	ELISA assay	↑ oligomeric α-synuclein in PD	Vivacque et al. [65]
	PD: 25 HS: 15	ELISA assay	↑ oligomeric α-synuclein/total α-synuclein in PD	Shaheen et al. [73]
	PD: 112 HS: 90 PSP: 20	ELISA assay	↑ oligomeric α-synuclein/total α-synuclein in PD	Vivacque et al. [66]
Oligomeric α-synuclein/total α-synuclein ratio	PD: 60 HS: 40	ELISA assay	↑ oligomeric α-synuclein/total α-synuclein in PD	Vivacque et al. [65]
	PD: 25 HS: 15	ELISA assay	↑ oligomeric α-synuclein/total α-synuclein in PD	Shaheen et al. [73]
	PD: 112 HS: 90 PSP: 20	ELISA assay	↑ oligomeric α-synuclein/total α-synuclein in PD	Vivacque et al. [66]
	PD: 201 HS: 67	Luminex assay Western blot Magnetic bead- based Luminex assay	↑ oligomeric α-synuclein/total α-synuclein in PD with disease progression	Kang et al. [72]
Deglycase-1 protein (DJ-1)	PD: 24 HS: 25	Western blot	No significant difference in DJ-1 between PD and HC	Devic et al. [74]
	PD: 16 HS: 22	ELISA assay	↑ DJ-1 in PD ↑ total protein in PD	Masters et al. [75]
Oxidative stress markers – heme oxygenase-1 (HO-1)	PD: 58 HS: 59	ELISA assay Western blot	↑ HO-1 in PD	Song et al. [78]
Acetylcholinesterase (AChE) and total salivary protein (TP)	PD: 30 HS: 49	Ellman colorimetric method	↑ AChE activity in PD ↑ AChE/TP ratio in PD No correlation between AChE, AChE/TP ratio and UPDRS scores	Fedorova et al. [79]

↑: increasing; ↓: decreasing; α-synuclein: alpha-synuclein; PD: Parkinson’s disease; HS: healthy subjects; PSP: progressive supranuclear palsy; UPDRS: Unified Parkinson’s Disease Rating Scale.
